# DieTryin: An R package for data collection, automated data entry, and post-processing of network-structured economic games, social networks, and other roster-based dyadic data

**DOI:** 10.3758/s13428-021-01606-5

**Published:** 2021-08-02

**Authors:** Cody T. Ross, Daniel Redhead

**Affiliations:** Department of Human Behavior, Ecology, and Culture, Max Planck Institute for Evolutionary Anthropology, Leipzig, Germany

**Keywords:** Social networks, Behavioral economics, Social relations, Economic games, Automated data entry, Peer report data

## Abstract

Researchers studying social networks and inter-personal sentiments in bounded or small-scale communities face a trade-off between the use of roster-based and free-recall/name-generator-based survey tools. Roster-based methods scale poorly with sample size, and can more easily lead to respondent fatigue; however, they generally yield higher quality data that are less susceptible to recall bias and that require less post-processing. Name-generator-based methods, in contrast, scale well with sample size and are less likely to lead to respondent fatigue. However, they may be more sensitive to recall bias, and they entail a large amount of highly error-prone post-processing after data collection in order to link elicited names to unique identifiers. Here, we introduce an R package, DieTryin, that allows for roster-based dyadic data to be collected and entered as rapidly as name-generator-based data; DieTryin can be used to run network-structured economic games, as well as collect and process standard social network data and round-robin Likert-scale peer ratings. DieTryin automates photograph standardization, survey tool compilation, and data entry. We present a complete methodological workflow using DieTryin to teach end-users its full functionality.

## Introduction

In the psychological and sociological sciences, there is a keen history of developing and applying social network methods (Borgatti et al. 2009)—i.e., methods that quantify relationships (termed edges or ties) between individuals (termed nodes or actors). At the same time, there have been increasing calls to expand the general scope of data collection to include more technologically, geographically, and culturally diverse groups (Henrich et al. 2010; Nielsen & Haun, 2016; Nielsen et al. 2017; Broesch et al.,, 2020). Interdisciplinary and international social network research has enabled many broad-reaching empirical advances, e.g., about the forms and functions of social relationships (Cutrona, 1986), the evolutionary functions of emotion and inter-personal sentiments (Gervais & Fessler, 2017; Gervais, 2017), and the drivers of friendship (Dijkstra et al. 2013), personality (van Zalk et al. 2020), religion and religiosity (Power, 2017), and social status (von Rueden et al. 2019). Such methods have also been crucial to applied research, e.g., in public health (Holt-Lunstad et al. 2010; Smith & Christakis, 2008).

Likewise, in fields as diverse as evolutionary biology and cultural anthropology, there is an increasing uptake of field research concerning the properties, causes, and consequences of social networks and associated social-relational characteristics. Field researchers have conducted examinations of the mechanisms that govern the emergence and stability of social bonds (e.g., Silk et al., 2009; Rucas et al., 2006; Hooper et al., 2013; von Rueden et al., 2019), economic, physical, and emotional well-being (e.g., Crittenden & Zes, 2015; Koster & Leckie, 2014; Ready & Power, 2018; Pisor et al. 2020), knowledge and skill acquisition (e.g., Franz & Nunn, 2009; Hill et al. 2014; Barrett et al. 2017; Lew-Levy et al.,, 2020), disease transmission (e.g., Read et al. 2008; Salathé et al.,, 2010; Ready et al. 2020b), and a variety of other phenomena.

While the vast majority of human studies assess the structure of social relationships through survey and interview methods, recent studies (e.g., Rucas et al. 2010; Gervais, 2017) have introduced novel and valuable methodologies for administering network-based experimental economic games (see also Pisor et al.,, 2020). These newly-developed games marry the strengths of experimental economic games to reliably measure preferences (Henrich et al. 2001, 2005) and the strengths of socio-relational data to measure how individual-specific characteristics and inter-personal relationships structure behavior in real-world networks—i.e., they extend economic games from measuring only the effects of a focal individual on allocation decisions, and allow measurement of recipient- and dyad-specific effects.

Classical economic games, like the Dictator and Ultimatum games (Henrich et al. 2001, 2005), are able to measure specific preferences of focal respondents—e.g., how willing they are to give resources to an anonymous same-community recipient/alter at a cost to themselves. While cross-cultural comparisons of such characteristics have been hugely influential, the simplistic game structure precludes investigations of how variation in *focal, alter, and dyadic* characteristics, might affect site-level preferences for specific behaviors, e.g., giving/keeping resources. The three network-structured economic games (termed RICH games, or recipient identity-conditioned heuristics games) introduced by (Gervais, 2017) generalize classical economic games for studying cooperation, exploitation, and punishment, and measure dyad-level behaviors using a full-community photograph roster. According to Gervais (2017), “...these RICH economic games tap the norms and motives that regulate enduring social relationships” [p. 127]; in other words, they allow researchers to study not just the behavioral preferences of focal respondents towards anonymous alters, but additionally how focal and alter characteristics (e.g., age, sex, wealth, religion, social network centrality, prestige, dominance, etc.), as well as dyadic characteristics (e.g., kinship, friendship, group-membership, etc.), are related to dyadic behaviors like resource transfers, exploitation, and spiteful punishment—permitting much more nuanced investigation of the drivers of cross–cultural variation in inter-personal behavior.

Despite their enormous promise for testing a wide array of questions in cross-cultural psychology, economics, and anthropology, full-community roster-based methods for collection of experimental economic game data and other dyadic measures have not been used as widely as other data-collection methods. Researchers studying social networks and inter-personal sentiments in bounded or small-scale communities generally face a trade-off between the use of roster-based and name-generator-based methods. A core theoretical distinction between these data collection techniques is that roster-based methods solicit data from participants’ recognition of names or photographs, while name-generator-based methods solicit recall data (Ferligoj and Hlebec, 1999). Roster-based methods scale poorly with sample size, and can more easily lead to respondent fatigue (especially when administered verbally with names, rather than visually with a roster of photographs). However, they require less post-processing (i.e., record linkage and de-duplication), and generally yield higher quality data, as recognition memory is generally recognized as more accurate than recall memory. Roster-based methods have been shown to capture a comparatively larger number of nominations (that also include the nominations collected via recall methods), and are less susceptible to the biases associated with recall (Bahrick et al. 1975; Hlebec, 1992; Hammer, 1984; Sudman, 1985). Name-generator-based methods, in contrast, scale well with sample size and are less likely to lead to respondent fatigue. However, they may be more sensitive to recall bias (Brewer, 2000), and they entail a large amount of highly error-prone post-processing after data collection, in order to link elicited names and nicknames to unique identifiers.

In order to make the use of powerful, roster-based research methodologies easier for field researchers working in large field-sites (e.g., small-scale societies or bounded communities), we introduce an R package, DieTryin, that allows for roster-based dyadic data to be collected and processed as rapidly as name-generator-based data. DieTryin was specifically developed to run network-structured economic games—e.g., Gervais' (2017) RICH games—as well as collect and process standard social network data and dyadic Likert-scale peer ratings. DieTryin automates photograph standardization, survey tool compilation, and data entry.

While other high-tech tools for social network data collection have been developed—e.g., Breadboard (Human Nature Lab, 2020a), Trellis (Human Nature Lab, 2020b), and Open Data Kit (ODK Team, 2020)—they generally rely on respondents interacting with electronics on the ‘front-end’. This can limit their usefulness to researchers like cross-cultural psychologists and anthropologists working in areas where the front-end use of electronics might be problematic. DieTryin instead allows for network-structured economic game data and social network data to be collected using simple, physical photograph rosters and game tokens (e.g., poker chips), and moves the high-tech functionality to the ‘back end’ where machine learning algorithms are used to automatically code edge-list data from photographs of token allocations.

In what remains of the paper, we provide: a) a brief overview of the benefits and limitations of different network measurement and sampling regimes, b) a review of data collection methods using a photograph roster, including a review of the RICH games methodology, and c) a description of the DieTryin R package and its unique functionality, including a step-by-step walk-through of the workflow for collecting and processing data from RICH economic games and other roster-based designs.

## Measuring social relationships

### Self-reported network data

Field studies of social networks typically employ one of two interview/survey techniques for collecting relational data. The first is the *name generator method*, which entails participants freely listing the names of other individuals within the community with whom they have a specific kind of relationship (Marsden, 1990). The second widely-used approach is a *roster-based design*, whereby the researcher generates a list of all members of a population and then asks each participant to report whether they have a specific kind of relationship with each-and-every individual on the roster (Marsden, 2005). In exceptional cases, social networks can also be created from long-term ethnography or observation (see Ready et al.,, 2020a; DeTroy et al. 2021), focal or scan sampling (see Altmann, 1974; Amato et al. 2013), and direct GPS tracking or proximity detection (see Davis et al. 2018; Wood et al.,, 2021). Each of these methods carries their own costs and benefits.

The principle benefits of the name generator method are that: (i) it is comparatively fast to implement—i.e., the time burden of data collection and entry scales roughly linearly with sample size—and (ii) it captures extra-community ties. The principle costs of the name generator method are that: (i) there is more work on the back-end for researchers to match the names—and possibly nicknames—recalled by respondents with unique personal identifiers, especially in communities where it is common for several individuals to share the same first and last name (or nickname), and (ii) various forms of recall bias may impact data quality—e.g., some individuals may be more likely than others to forget real ties (Bell et al. 2007; Brewer, 2000), some alters with specific characteristics may be more likely to be remembered or named by others in the community (Marin, 2004), and differences across interviewers in terms of personality type and data collection style can influence the number of names elicited (Harling et al., 2018). These costs and benefits are more or less inverted in roster-based designs.

In roster-based designs: (i) data collection and entry burden scales with the square of sample size, and (ii) the method fails to easily capture ties to extra-community individuals not appearing on the roster. However, there are some benefits: (i) there is no work on the back-end to link names to personal identifiers, as the roster can be constructed to differentiate between individuals with the same name a priori, and (ii) recall bias is minimized by presenting each focal respondent with a prime—be it a name or a photograph—of each alter in the community. While these trade-offs need to be considered on a case-by-case basis, DieTryin aims to increase the feasibility of roster-based methods by automating the data collection and entry process so that the time burden scales linearly with sample size.

### Experimental network data

In an attempt to reduce some of the potential biases (e.g., recall bias, researcher demand bias, social-desirability bias, etc.) associated with self-report techniques for capturing social networks, social scientists have recently advanced network-based extensions of several classical games developed in experimental economics (Centola, 2010; Kearns et al. 2009; Suri and Watts, 2011). Such experiments are generally devised to test specific predictions from game theory, and are frequently conducted among relatively homogenous samples of undergraduate students in laboratory settings using networked computers or administered as online network-based games (e.g., Colman, 2016; Charness et al. 2014). Within these paradigms, researchers provide monetary incentives for participation and pose a specific social problem between a dyad or group, where there may be competing interests, and where participants’ decisions dictate their financial payoffs.

These network-based experimental games generally have high internal validity and have made many important contributions to the understanding of human preferences and behavior (e.g., Cassar, 2007; Rand et al. 2014; Rand et al. 2011; Ohtsuki et al. 2006; Suri & Watts, 2011). However, these paradigms have also been criticized for their potential lack of generalizability and external validity (Hagen & Hammerstein, 2006). In response, experimental game paradigms are now being administered in naturalistic settings (e.g., Rucas et al.,, 2010; Gervais, 2017; Pisor et al.,, 2020) to test their validity in real-world contexts.

### Sampling

To successfully capture relational data with either self-report or experimental measurement instruments, researchers generally need to collect data about the social relationships between most, if not all, individuals within a focal community. This is important because the statistical properties of higher-order network characteristics can be very sensitive to even a relatively small proportion of missing data (Granovetter, 1976; Frank, 2005). In most contexts, researchers will restrict their samples and strategically collect data from participants of a specifically defined group, often based on: (i) residence within a delineated geographic area (e.g., residents between two rivers), (ii) membership in a specific group (e.g., children in classrooms), or (iii) participation in mutually-shared events (e.g., ‘regulars’ at a bar or pub), and only consider the reported relationships within the sample (Laumann et al. 1989). Only in certain cultural contexts, where participants reside in relatively small and geographically-isolated populations, is capturing ‘complete’ population networks feasible.

## A complete workflow with DieTryin

In what follows of the paper, we provide step-by-step instructions on how to use DieTryin to prepare, collect, and process the data resulting from network-structured economic games and other dyadic data collection methodologies. We also provide an accessible tutorial workflow at: https://github.com/ctross/DieTryin, which contains additional annotated R code and example photograph datasets. Our tutorial trains end-users to run a full workflow using DieTryin to prepare for, conduct, and manage a real-world, roster-based social network study. Bug-reports, feature requests, and other relevant comments should be made through GitHub, where the package will be maintained.

**Fig. 1:**
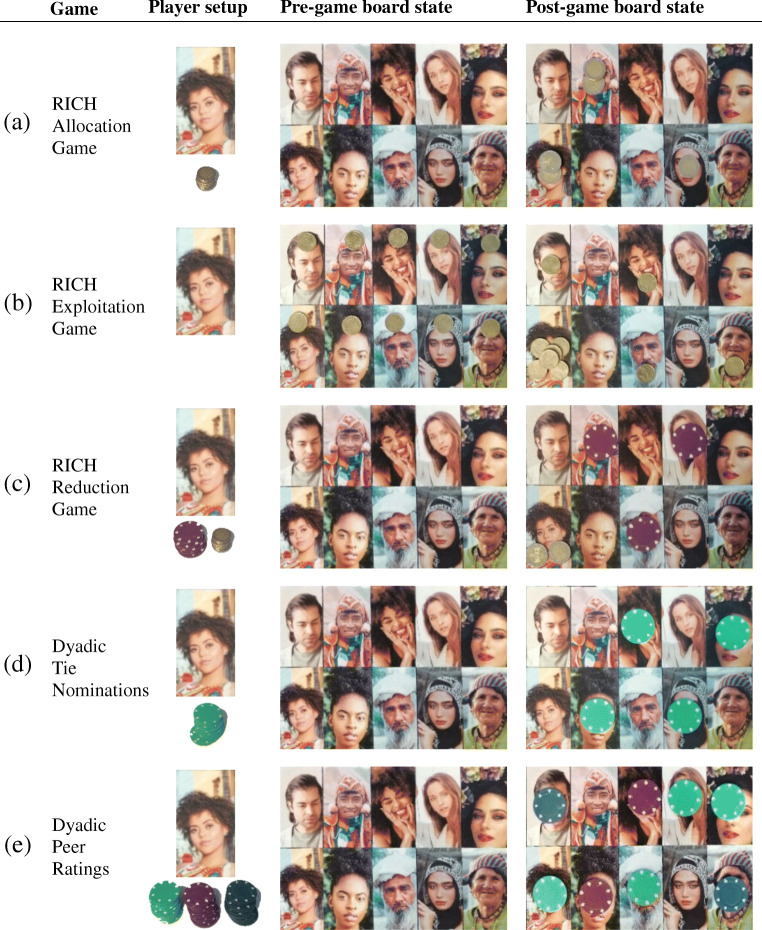
Here we outline methods for data collection using a photograph roster. **a** In the RICH allocation/giving game, each player is given a stack of coins and presented with a blank game-board; the player can give coins to anyone in the community by placing any number of coins on that person’s photograph. Coins not allocated to others are kept for the self by placing them on the focal’s own photograph. **b** In the RICH exploitation/taking game, each player is presented with a game-board in which all alters have a pre-existing allocation; the player can exploit others by taking the coin(s) off of their photographs and transferring them to the focal’s own photograph. **c** In the RICH costly reduction/punishment game, each player is presented with an empty game-board and a stack of coins; the player can keep these coins by placing them on their own photograph, or use these coins to purchase tokens that can be used to reduce the payout of any alter on the game board. **d** To define dyadic ties, a focal player is given a large stack of colored tokens and asked to place a token on each individual with whom they have a specific kind of social tie (e.g., their close friends). **e** To measure dyadic peer ratings, a focal player is first given several large stacks of colored tokens. The respondent is then told, for example, to place a blue token on very trustworthy people, a green token on very untrustworthy people, a purple token on people of average trustworthiness, and no tokens on individuals whom they do not know well enough to rate

Although DieTryin has much functionality specifically tailored to Gervais’ (2017) RICH economic games, it is considerably more general and can be used to facilitate collection, entry, and processing of round-robin peer ratings and roster-based self-report measures. These measures are widely used in psychology and other related disciplines to capture perceptions of peers—e.g., their personality, status, dominance, or behavioral profiles—as well as reports of dyadic characteristics—e.g., church co-membership, directed food/money transfers, or kin relationships. In Fig. 1, we provide a brief visual overview of each of these data collection methods.

### Installation and setup

Much of the functionality of DieTryin is made possible by R (R Core Team, 2019) and the imager (Barthelme, 2019), MASS (Venables & Ripley, 2002), and xtable (Dahl et al. 2019) packages and their dependencies, as well as by the LATE X software system. The user must install these programs in order to reproduce our workflow.

Installation and loading of DieTryin is then simple: just run three lines of code from R: 
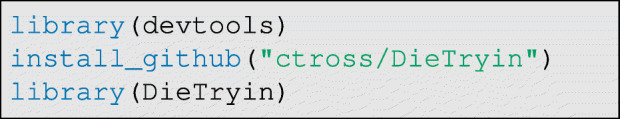


Next, we set a path to where we will save all of the files related to our project, and initialize a directory structure there: 
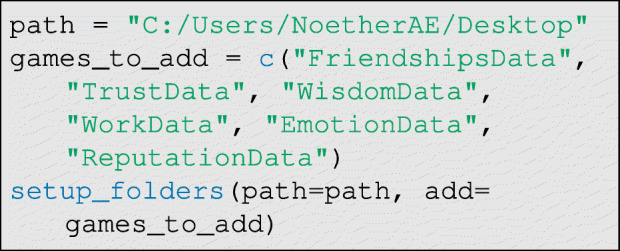


This step creates a directory—titled RICH—with all of the sub-folders needed to organize the workflow. The standard function call—i.e., setup_folders(path)—sets up data storage only for the three RICH games, but additional folders—for example, to store data on friendship ties—can be added as well, by setting: add=games_to_add, where games_to_add is a vector of folder names that should be created.

### Standardization of photographs

**Fig. 2:**
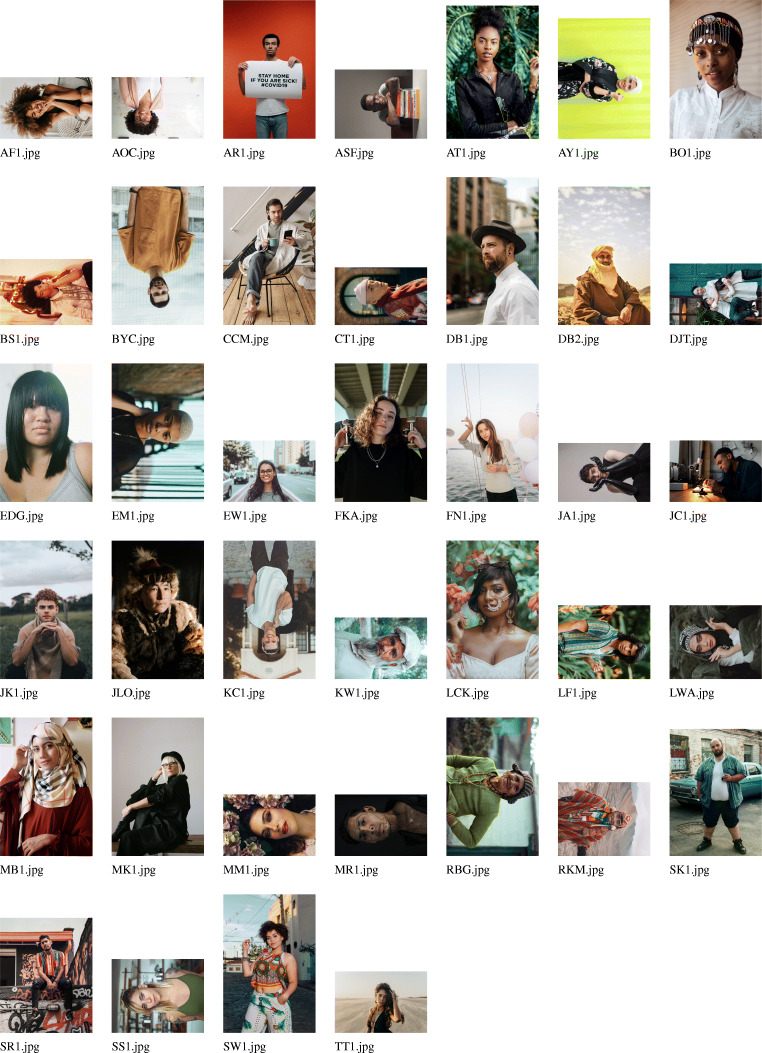
Example raw photographs—corresponding to fictive respondents from a fictive field-site—prior to processing with DieTryin. Photographs vary in terms of size, aspect ratio, orientation, zoom, and centering. In large field-sites, manual adjustment of these variables for each respondent photograph using standard tools can be time consuming. DieTryin uses an R script to automate image standardization. With a single line of code, a database of images can be rapidly processed. Pexels (Bruno et al. 2020) has made these sample images available for free and open use. Sources and credits for each photo are included in the supplementary materials

The next step of the workflow is to create the photograph roster used in data collection. Once a full sample of informed and consenting research participants is selected, photographs of each participant can be taken, and the research process started. The raw photos of all respondents who will take part in the network-structured economic games and/or do a social network interview should be copied into the RICH/RawPhotos directory. These should be jpg-formatted images. The filenames must include the unique identifier (ID) codes for the respondents: e.g., AOC.jpg, KW1.jpg, JLO.jpg. These filenames should all be of the same character length and contain a letter as the first character. Raw photographs, however, will frequently vary in terms of size, aspect ratio, orientation, zoom, and centering (e.g., see the example database of raw photographs in Fig. 2), and response validity can be improved by standardization. In large field-sites, manual adjustment of respondent photographs can be time-consuming. To facilitate the standardization of respondent photographs, DieTryin includes a function that largely automates the process of image standardization.

Once the full set of respondent photographs are copied-and-pasted into the RICH/RawPhotos folder, the full directory of images can be rapidly processed with a single line of code: 
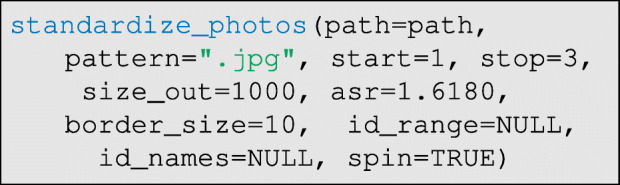


The arguments to standardize_photos are intuitive: pattern is simply the file extension (accepting either ".jpg" or ".JPG"), start and stop give the locations of the first and last letters of the unique identifier in the filename of each photograph, size_out gives the width of the exported photograph in pixels, asr gives the aspect ratio, and border_size gives the number of pixels on the boundary of each photograph to be colored black.

In normal contexts, the standardize_photos function is programmed to run over all photographs in the RICH/RawPhotos directory. However, if only a small range of photographs (or a single photograph) needs to be processed, then this can be specified. For example, by changing id_range=NULL to id_range=c(1:5), only the first five photographs in the directory will be processed. Likewise, changing id_names=NULL to id_names=c("BYC", "FN1") instructs the program to only process the photographs of the individuals with those specific ID codes. This can be useful to correct user-introduced errors in specific processed photographs.

**Fig. 3:**
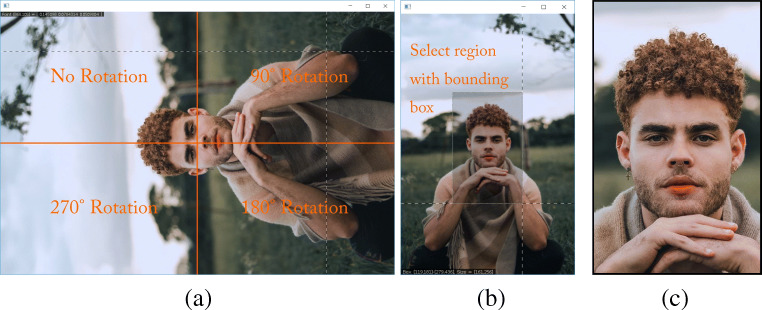
Image standardization using a fictive resident from our fictive field-site. **a** Photograph rotation is accomplished by clicking in one of four quadrants. **b** Standardization of facial area is done by dragging and dropping a rectangular bounding box. **c** Result is a standardized photograph. The R script loops through all raw photographs in the database and saves all standardized photographs in a dedicated folder

**Fig. 4:**
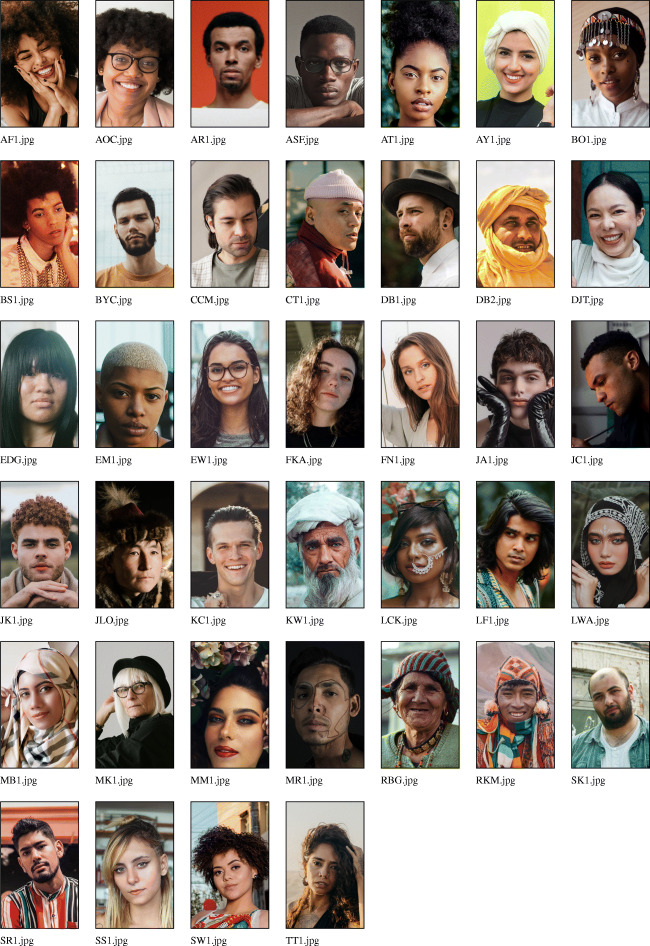
Example standardized photographs—corresponding to fictive respondents from a fictive study site—after processing with DieTryin. Photographs are now of a fixed size, aspect ratio, orientation, zoom, and centering

Finally, the argument spin=TRUE can be set to FALSE, in order to skip the photo-rotation step—discussed below—if all photographs are already correctly oriented. Otherwise, for each photograph in the RICH/RawPhotos directory, standardize_photos will open a two-step process. First, the raw image will be displayed as it is stored by R. The user must then click in one of four quadrants on the displayed photograph: a click in the upper-left will apply no rotation, a click in the upper-right will apply a 90^∘^ clockwise rotation, a click in the lower-right will apply a 180^∘^ rotation, and a click in the lower-left will apply a 270^∘^ clockwise rotation (see Fig. 3a). Next, the correctly oriented picture will be displayed. The user must then click and drag a bounding-box giving the approximate area to be used as the final processed photograph (see Fig. 3b). Repeat these steps for each photo. Each standardized photograph will be saved to the RICH/StandardizedPhotos directory, where they can be checked by the user for quality (see Fig. 3c). Figure 4 presents the photographs from Fig. 2 after applying the standardization procedure.

### Building the survey tool

Once the respondent photographs are standardized, they can be printed out, laminated, and arranged on boards. To randomize the positions of photographs on the game boards and generate a survey tool that can be used to efficiently record data, DieTryin provides the build_survey function. Replicable randomization of photographs is done using the seed argument. However, if a specific order of photographs is desired, this can also be specified as we show below. Then, a single line of code will generate a LATE X document and compile it to PDF: 
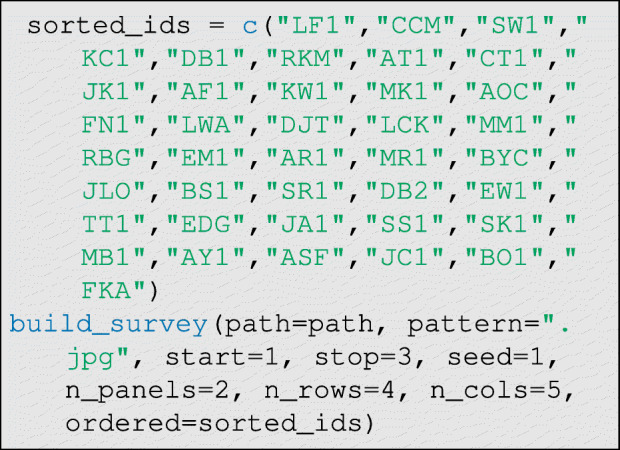


The seed argument gives the starting value for the random number generator used to randomize the order of photographs, the n_panels argument gives the total number of panels/boards of photographs to be displayed, and the n_rows and n_cols arguments give the number of rows and columns of photographs per panel/board. It is generally important to randomize the ordering of photographs on the panels a few times over the course of a single field-season, especially when conducting the economic games. By changing the seed argument, the order of photographs on the survey tool can be changed. Later, this same seed value will be passed to the data-entry functions in order to facilitate data entry. If the user wishes to pass in a specific order of photographs, a vector of IDs can be passed in via the ordered argument, which will take precedence over seed, otherwise set ordered=NULL to randomize order.

**Fig. 5:**
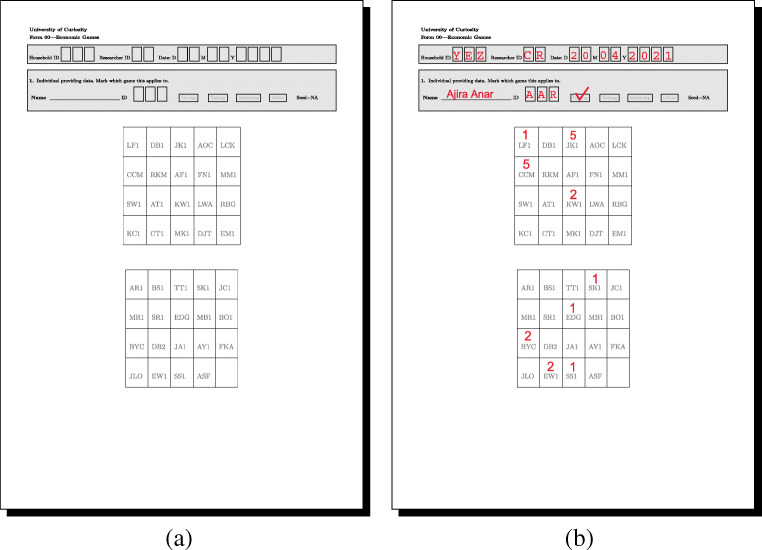
This PDF can be used to record RICH game outcomes and other dyadic measures. Personal identifiers are automatically entered into the tables by DieTryin using the ID codes of the standardized photographs. Frame (**a**) illustrates the exported survey tool. Frame (**b**) illustrates how data on directed coin allocations may be recorded

Figure 5 illustrates an example survey tool compiled by DieTryin. Frame (a) illustrates the exported survey tool. Frame (b) illustrates how data on directed coin allocations may be recorded using the survey tool. Note that the header of the survey tool can be customized by editing the header.txt file in the RICH/Survey directory before running the build_survey function.

### Assembling the game boards

**Fig. 6:**
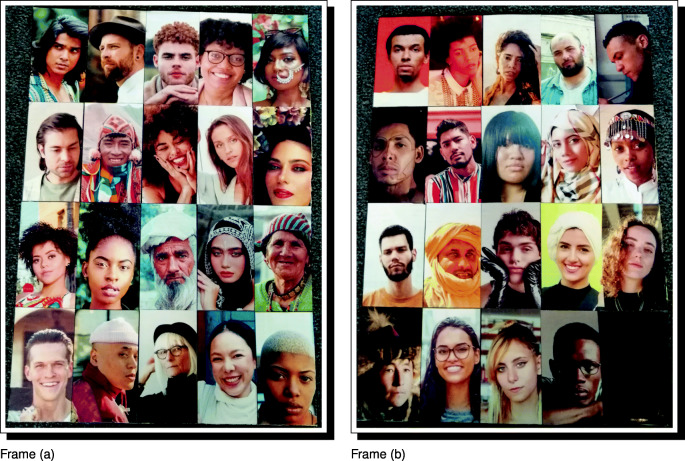
Two physical game boards, filled with standardized and randomized recipient photographs. The order of panels can be randomized and recorded between repondents, and the order of photographs on the boards can be randomized as needed, by repeating the survey building code using different seed values

Once the survey tool is compiled, the standardized respondent photographs can be arranged on the game boards in the same order as is the survey. For even moderately large field-sites, it is common to need multiple boards/panels. Figure 6 illustrates a layout of 39 respondents distributed over two panels.

For field-sites up to around 300 respondents, it can be feasible to run RICH games and other forms of dyadic data collection using a photograph array of all residents. For larger field sites, it might be necessary to create a large number of panels, and then present only a random subset of these panels to each focal respondent, randomizing the set of panels between respondents. This ensures balanced potential payoffs across all recipients in the field-site, without requiring each focal to make a decision with respect to each and every alter. Balanced potential payoffs are important for ensuring that all recipients feel that everyone in the community has equal and fair access to the possible benefits from the games. Another possible strategy is to run the economic games and other forms of dyadic data collection independently within sub-communities of feasible size. Either of these methods comes with inferential trade-offs that must be considered in light of the research goals.

### Manually entering data

For some kinds of data, especially data from the allocation or costly reduction games—where multiple coins can be allocated to each person—it is often best to enter data manually using the enter_data function: 
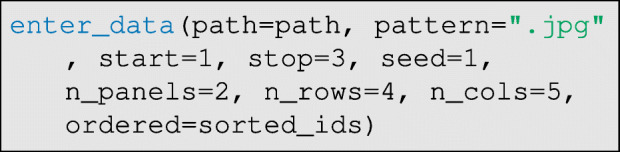


The function will open a pop-up window in R, and start a two-step data entry process. In the first step, the header data (i.e., name, date, and ID code) is entered. In the second second step, the network data is entered. If one is entering data from the first game of a given respondent, then type: Y, and the header data will be cleared. Otherwise, if one wants to save some time and carry the header data forward (e.g., if one is entering data on a second game from the same person), type: N, to carry the prior header data forward.

**Fig. 7:**
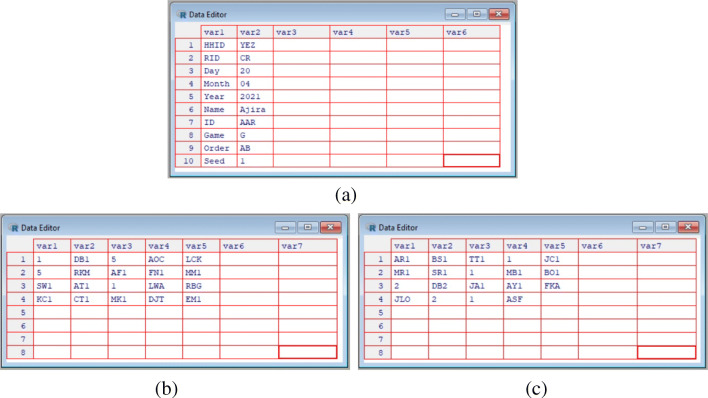
The manual data entry workflow. Frame **a** illustrates the required header data. Frames **b** and **c** illustrate coin allocation data correspoding to what is recorded in Fig. 5b. Personal identifiers are automatically entered into the tables, and are converted to zeros if not over-written by coin allocation data. In frame (**a**), HHID is household ID, RID is researcher ID, and ID is the personal ID of the focal respodent. Only ID and Game are required feilds, others can be left blank if desired

Now enter the header data as shown in Fig. 7a. Data must be supplied for ID and Game, but other entries can be left blank if desired. In the pop-up window, the entry for Game must take one of three special values for RICH games data (G for the giving/allocation game, L for the leaving/taking game, and R for the reduction/punishment game). Other question types can be given arbitrary names (corresponding to those supplied in the add argument of the setup_folders function). The argument Order gives the order of the panels as presented to the respondent, with A being the first panel in the PDF, B being the second, and so forth. The seed argument is prefilled during the call to the enter_data function. As long as the seed in the function call matches the seed printed on the PDF, the recipient IDs will be properly sorted and ready for data entry. Once the header data is entered, simply close the window in R; there is no need to save or hit ctrl+s.

A new pop-up window will open, as shown in Fig. 7b. Now, if coins were placed on a recipient’s ID, click on that ID code, type the number of coins placed, and then move on to the next ID. If no coins were placed on a recipient, just leave the ID code alone. There is no need to type in the zeros, as they will be filled automatically. Once the data are entered, simply close the window. Game data will be saved as a csv file in the appropriate Results directory. If errors are made in data entry, the resulting csv file can be edited by hand, or the enter_data function can be run again; if the function is run again, it will overwrite the previous (erroneous) version of the person-specific results file. The data in Fig. 7b and c correspond to the data recorded on the PDF in Fig. 5b.

When entering data for the three RICH games, DieTryin accepts only numerical values (i.e., the number of coins placed on a given alter). This requirement is relaxed when entering data from other question types: text strings can be used, for example, to define qualitative or categorical ties.

### Data compilation

After the data collection protocols have been completed by each participant and all data for each game have been entered by the researcher, then the individual-level csv files can be compiled into a single data-set and checked for basic data-entry errors. To do so, we run the compile_data function: 
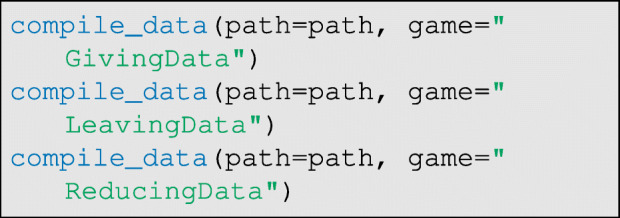


This will build two files for each game—a summary table and an edge-list—and store them in the Results folder. The summary table gives a basic self versus other coin allocation count, and checks that the sum of entries (i.e. the total number of coins/tokens appearing on the game board) is correct. If the checksum cell in the summary table for every respondent is not equal to the total number of coins used in a given game, then there was likely a mistake during data collection or data entry. Note, for example, the data entry error in Fig. 7, where the sum of entries is 19, even though 20 coins were distributed according to Fig. 5b. If the checksum for a given respondent is wrong, the corresponding game board photographs, surveys, and csv files should be checked and revised (in this example, the allocation on row 3, column 3, of game-board panel A must be changed from a 1 to a 2 to match Fig. 5b). The compile_data function must be rerun to integrate changes. Once all of the summary tables appear correct, we can continue with the workflow.

### Payout calculation

Once reliable edge-list data for all games have been compiled as above, payouts for each respondent can be calculated. To do so, we run the calculate_payouts function: 
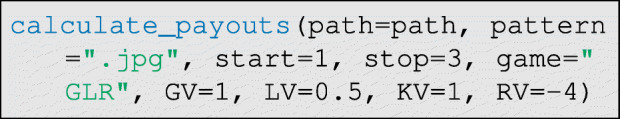


The argument game indicates which game data will be used in payout calculations. All combinations of G for the giving/allocation game, L for the leaving/taking/exploitation game, and R for the reduction/punishment game, are accepted: i.e., type "G", "L", "R", "GL", "GR", "LR", or "GLR". The argument GV gives the monetary value of the coins used in the giving game, LV gives the monetary value of the coins used in the leaving game, KV gives the monetary value of the coins kept for self in the reducing game, and RV gives the reduction value of the tokens in the reducing game. In the case that some recipients never appear as respondents—presumably due to temporary absence from the community—coins directed to these recipients are refunded to donors. The total payouts to each individual are displayed in R and saved as a csv file in the RICH/Results folder.

### Automatically entering data: Binary indicators

While RICH games data are often best entered manually, since there can be several coins allocated to each recipient, it can be useful to collect additional binary dyadic data: e.g., “With whom have you shared food in the last 30 days?” using the same photograph roster. By placing tokens of a known color on the photograph roster to indicate directed ties and then photographing the resulting game boards, a researcher can implement an automated data entry workflow with DieTryin.

**Fig. 8:**
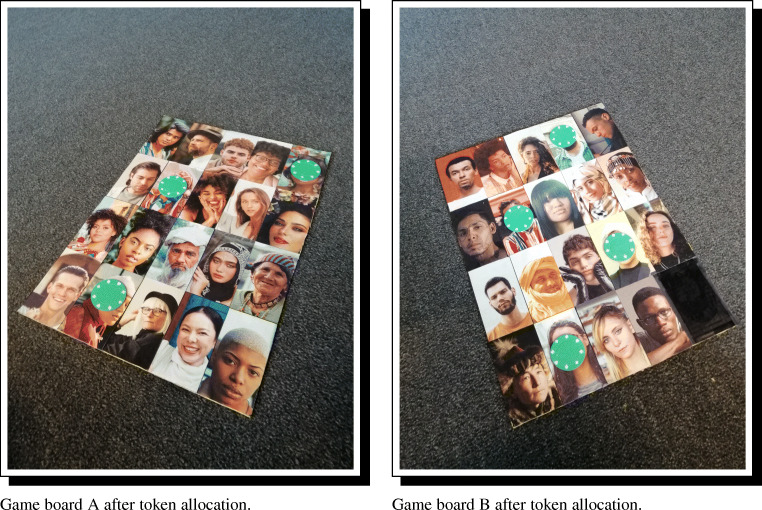
Photographs of the game boards after token allocations have be made to indicate dyadic ties. Note how the photographs may be rotated relative to center and affected by shearing. Although the amount of rotation and shearing depited here exceeds that which would be present in typical game board photographs taken by a careful researcher, even a small amount of rotation or shearing can make automatic classification difficult. To ensure accurate classification, DieTryin uses a two step process in R to first straighten and square the input photographs (Fig. 9), and then detect and classify token allocations (Fig. 10)

**Fig. 9:**
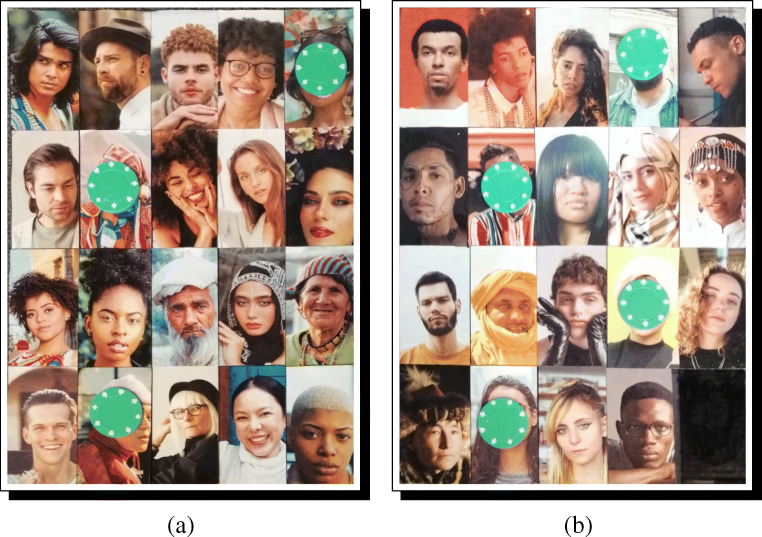
Simulated photographs of the game boards from Fig. 8 after applying an inverse transformation matrix to the skewed images. Note how the photographs are now cropped, unrotated relative to center, and no longer affected by shearing. At this point, individual recipient photographs are programmatically extracted from the array and analyzed using the automatic classification methods described in Fig. 10. DieTryin takes simple user input—point-and-click data on the corners of the game boards—and returns an edge-list of social ties (e.g., as in Table 1). Skew correction and token classification algorithms function under-the-hood, requiring no user input beyond identification of game board corners using a simple graphical user interface

Photographs of the game boards, however, normally suffer from rotation, skew, or shearing that can complicate automated data entry. To correct these visual distortions, DieTryin uses a two step process in R. First, the user must identify the corners of each game board with a click. Then, DieTryin will identify the camera position relative to the game board, and apply an algorithm that corrects any distortions. See Fig. 8 for unprocessed photographs of the game boards and Fig. 9 for photographs of the game boards after algorithmic distortion correction and cropping.

**Fig. 10:**
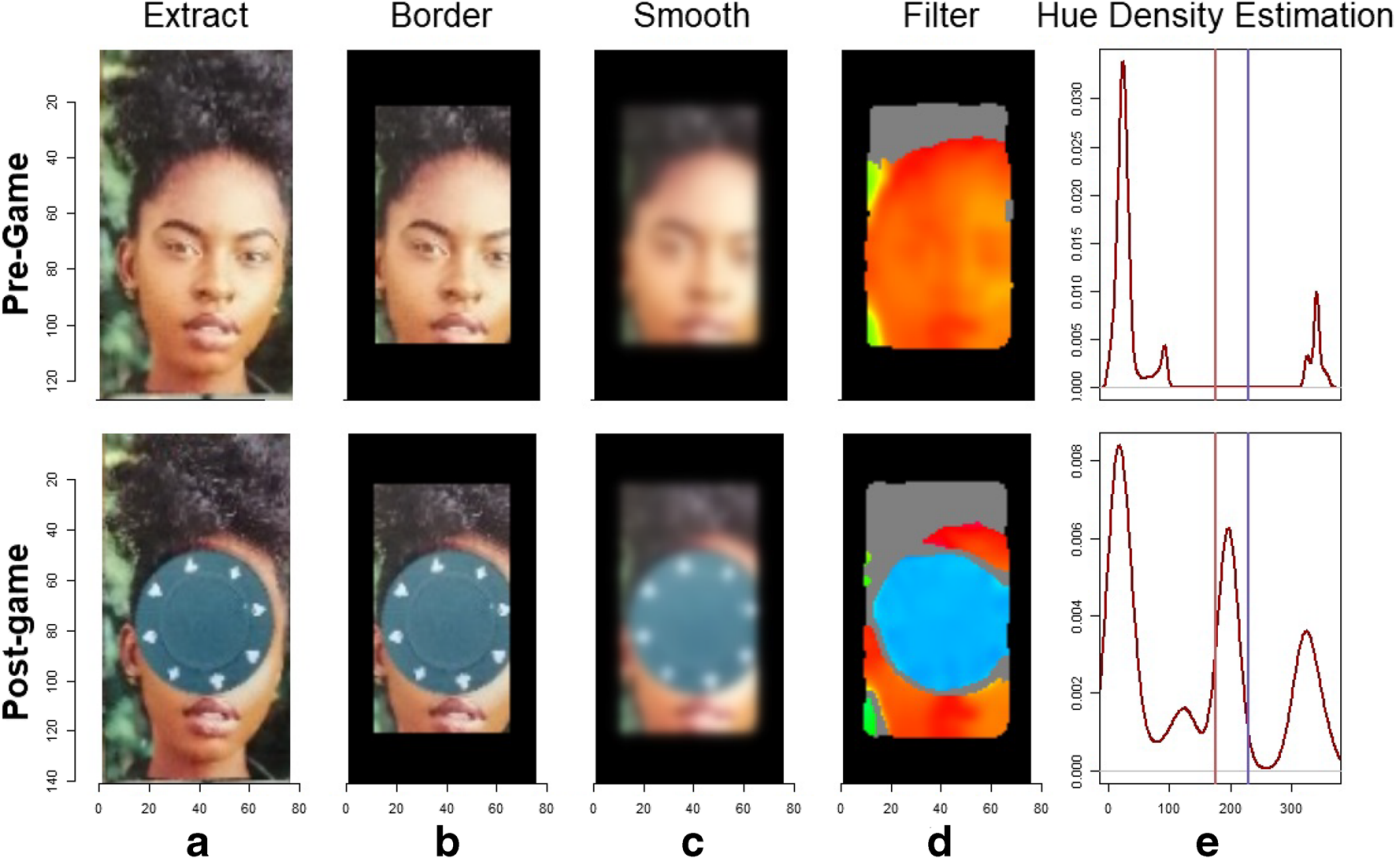
The photograph processing workflow implemented by DieTryin. In step A, recipient photographs are extracted from the main array. In step B, a border width is excised to minimize the influence of clothing and background color. In step C, a Gaussian blur is applied to smooth out high-contrast regions. In step D, the standard RGB-image is converted into an HSL-image and threshold filters are applied based on saturation and luminosity layers; the resultant hue layer is then extracted. Finally, in step E, the density distribution (shown here in dark red) of hue values is calculated for each image and integrated between lower and upper hue thresholds (shown here in vertical light red and light blue bars). If the difference in area under the curve (and between hue thresholds) in the pre- and post-allocation images exceeds a threshold parameter, then a directed tie is coded as present. This process is repeated for each token color and for each respondent photograph; the resultant color-labeled edge-list is then returned to the user

Next, once undistorted images have been produced, they are automatically fed into a classification algorithm. Under-the-hood, each individual respondent photograph is extracted from the overall image array using the dimensions of each panel, and the number of photograph rows and columns it contains. The recipient’s ID code is mapped onto this image. For each recipient, pre- and post-allocation photographs are then analyzed and compared. Figure 10 provides an outline of the method used to identify the presence/absence and color of tokens.

For a user to implement automatic data entry, all photographs of a given respondent’s game boards must be pasted into the RICH/ResultsPhotos directory. To account for variation across respondents in the lighting of the game boards, there should also be a photograph of each panel for each individual with no tokens placed (the control condition). The file-names of these images must be formatted as: Blank_AAR_A.jpg. The first string is the word Blank followed by an underscore, then the respondent’s ID code, then another underscore, and finally the board/panel ID. For each additional question/game, another set of photographs is provided. These file-names must be formatted as: Game_AAR_A.jpg, but in this case, Game can be replaced with an arbitrary text string.

Then, to speed up the classification algorithm, photographs of the game boards can—optionally—be resized to a smaller dimension: 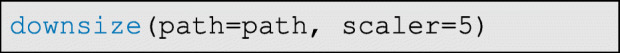


This line of code will copy all game board photographs, downsize them by a factor of 5, and save them in a new folder. If the files do not need to be resized, set scaler= 1.

Next, the user must pre-process the images. The pre_process function opens an interactive window that displays each photo array. The user must click the top-left corner of the photograph array, then the top-right, bottom-right, and bottom-left, in that order. This provides DieTryin with the location information needed to crop-out only the photograph array, and correct any rotations or distortions. The user will need to process the blank boards and the boards for at least one other question/game. First run: 
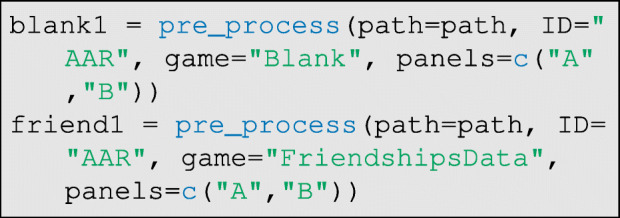


where game is the game ID code, and panels are the board/panel ID codes. Then, we can wrangle all of the above data for each game into the list structure needed for the classifier: 
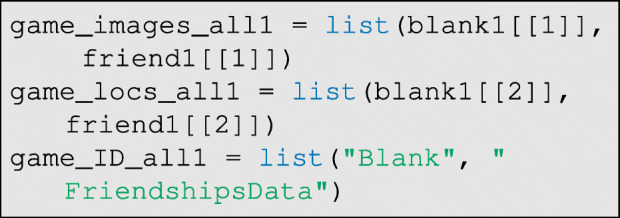


Each of the lists above can be extended as needed. See GitHub for more details on batch processing/vectorization across several games from the same respondent.

Now we can run the data entry function. This is the most computationally-intensive step in the data entry process: 
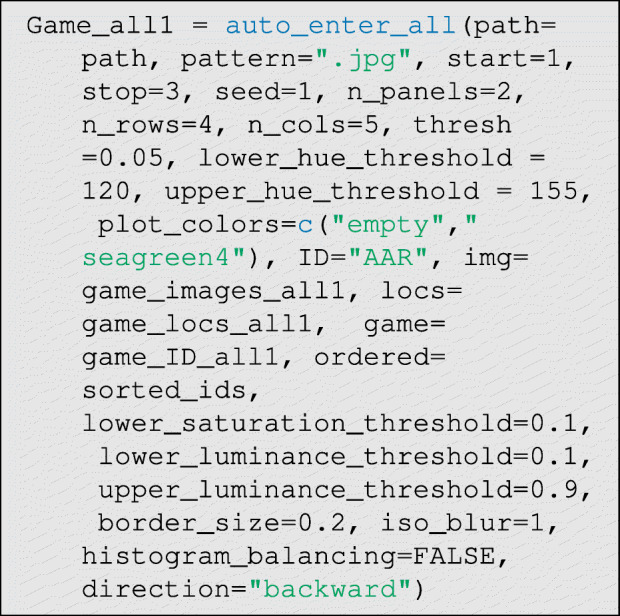


The function defaults generally work well, so only the hue thresholds should need to be set by the user in most cases. There are three main parameters that control classification performance: thresh∈ (0,1), which controls how much the percent difference in hue density must diverge between control and treatment cases for a tie to be declared, and lower_hue_threshold∈ (0,360) and upper_hue_threshold∈ (0,360), which give the lower and upper bounds of the hue range corresponding to the token color (see Fig. 10 step E). To identify good hue threshold values for a given token color, it is helpful to use a color picker app that can interactively plot the HSL (Hue, Saturation, and Luminescence) values for the pixels corresponding to tokens on a given photograph. We provide a simple interactive application in R—via the function: get_hue(file.choose())—for this purpose, but many online tools are also available.

Additionally, there are several other more technical parameters that can be modulated from their defaults to control classification: lower_saturation_threshold∈ (0,1) controls the limit of saturation at which hue values are excluded from measurement (because they are essentially grey) in step (D) of Fig. 10, lower_luminance_threshold∈ (0,1) and upper_luminance_threshold∈ (0,1) control the values of luminescence at which hue values are excluded from measurement (because they are essentially black or white, respectively) in step (D) of Fig. 10, iso_blur controls the width (in pixels) of the isoblur applied in step (C) of Fig. 10 (a value of 0 turns off isoblur), border_size∈ (0,1) controls the width (in percent) of the excluded border in step (B) of Fig. 10, histogram_balancing is an indicator variable for whether histogram balancing should be applied to enrich the photographs prior to processing, and finally direction∈{"forward", "backward"} indicates whether the distortion correction algorithm should be run in forward or backward mode. Forward mode is fast but produces lower-quality images (that may nevertheless permit perfect classifications), and backward mode is slower but produces higher-quality corrected images (see imager documentation for further details about these modes).

Running the above steps on the images presented in Fig. 8, yields an edge-list as an output (see Table 1). The classification model with default settings generally works well, but performance can be sensitive to input parameters, including the legal range of hues attributable to each token, and the required divergence in hue density between control and treatment photographs. These parameters can be optimized by the user prior to fieldwork using simulated allocations. Tokens of the cool hues like green, blue, and purple are generally easier to correctly classify, as they are less likely to overlap with skin hue than tokens of warm colors, like red or orange. Surprisingly, use of black-and-white recipient photos can decrease classification accuracy, since the hue of values of such photos in the control condition can vary a lot based on ambient lighting.

**Table 1:** The directed ties present in Fig. 11, as inferred by the classifier. Inferred ties should always be checked visually, by plotting these classified ties back onto the raw images

PID	AID
AAR	RKM
AAR	CT1
AAR	LCK
AAR	SR1
AAR	EW1
AAR	SK1
AAR	AY1

**Fig. 11:**
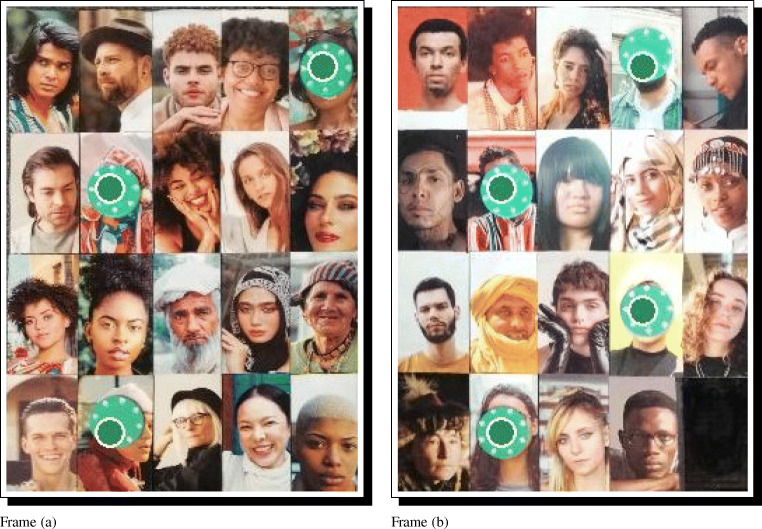
Predicted dyadic ties corresponding to the data in Table 1 overlaid on the relevant game-board photographs. Green points indicate where the model has predicted that green tokens have been placed. In this case, we see that the model has correctly classified all true ties, and has not erroneously classified any non-ties. The model parameters must generally be tuned by the user before perfect classification is achieved. Automatic classifications should always be visually checked before the data are used to make scientific inferences

To check that the inferred edge-list is correct, the user can run the check_classification code: 
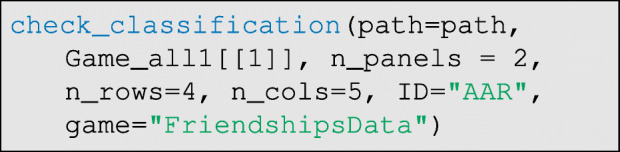


This code will map the inferred ties back on to the photograph array, and save the resulting output in the RICH/ClassifiedPhotos folder, where each panel can be checked for accuracy. See Fig. 11, which demonstrates perfect classification in this test. More broadly, we repeated this process with 26 different allocations of 20 tokens per round across the 39 possible recipients, and find that 520 of 520 true ties were correctly identified, and that 0 of 494 non-ties were mistakenly classified as ties. In other words, with minor tuning of essential parameters, excellent automatic classification is possible using DieTryin.

If the inferred classification of tokens is good, header data can be appended and the results saved as a csv file using the annotate_data command: 
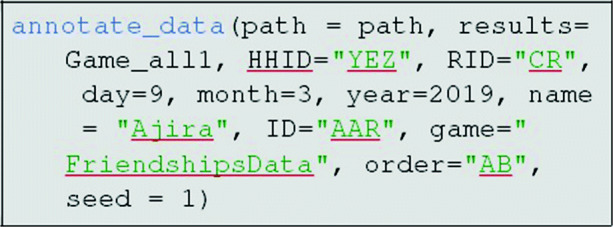


This function will export a data file of the same format as the standard enter_data function. Once all data are entered, the final edge-list can be compiled in the same way as was done for manual entry: 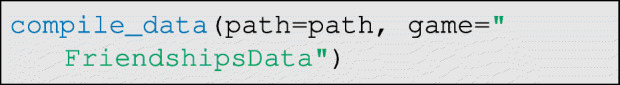


### Automatically entering data: Likert-scales

**Fig. 12:**
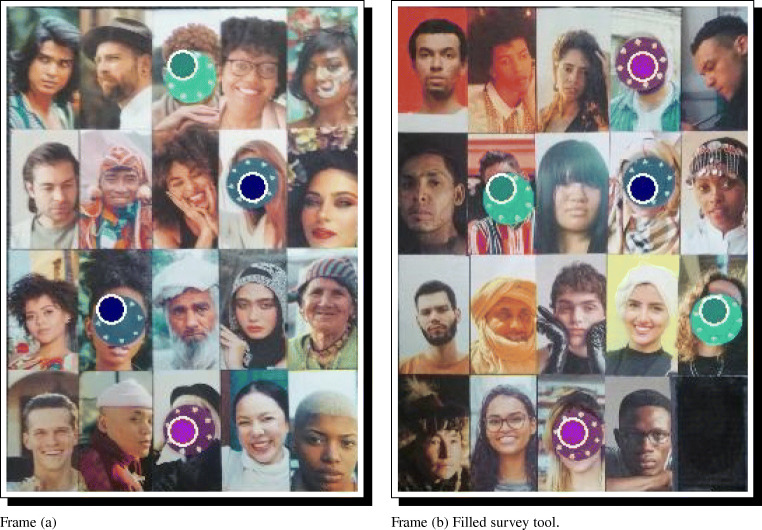
Predicted dyadic ties corresponding to the data in Table 2 overlaid on the relevant game-board photographs. Colored points indicate where the model has predicted that tokens of the same color have been placed. The model performs well for blue, green, and purple tokens, but tokens of red or orange hue can be hard to classify, as these hues are more likely to conflict with skin hue. As stated before, automatic classifications should always be visually checked to ensure that there are no errors introduced by the classification algorithm

In addition to simple binary directed ties, researchers often desire dyadic peer ratings between respondents using Likert-scales. DieTryin supports dyadic Likert-scale ratings through the use of tokens with different colors: e.g., blue = − 1, purple = 0, green = 1, blank = NA. Up to five token colors are supported. Data are prepared as before, but now we run: 
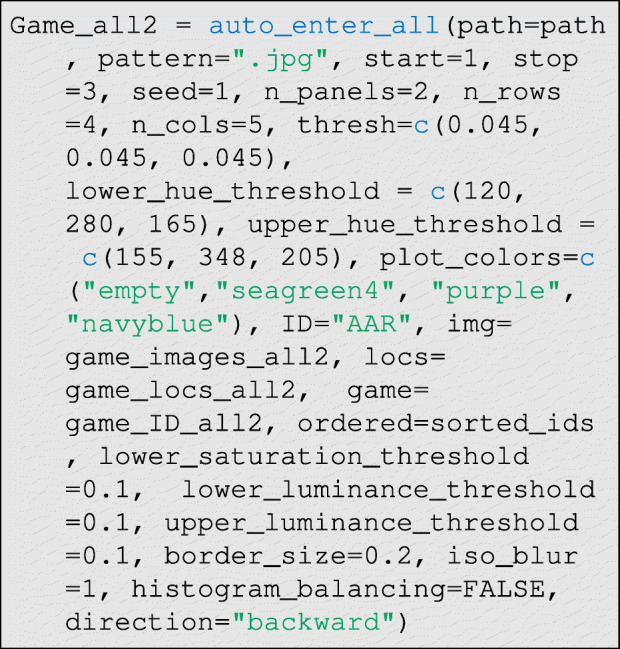


where a vector of lower and upper hue thresholds are supplied, and matched to a vector of color codes. The check_classification code can be run as before, with no modifications. In Fig. 12, for example, we attempt to classify categorical ties using three token colors. All ties are again correctly classified (see Table 2). More broadly, we repeated the process with 26 different allocations of 9 tokens of each of 3 colors per round across the 39 possible recipients, and find that 702 of 702 true ties were correctly identified, that 0 of 312 non-ties were mistakenly classified as ties, and that no tokens of one color were mistakenly classified as a different color.

**Table 2:** The directed ties present in Fig. 12, as inferred by the classifier. Note that the edge list now has a column giving token color which can then be mapped to ordered categories in R

PID	AID	Token Color
AAR	AT1	navyblue
AAR	FN1	navyblue
AAR	MB1	navyblue
AAR	MK1	purple
AAR	SS1	purple
AAR	SK1	purple
AAR	JK1	seagreen4
AAR	SR1	seagreen4
AAR	FKA	seagreen4

### Automatically entering data: Input-free coding

In the examples above, user input is required to identify the corners of the game boards in haphazardly taken photographs. If data are collected using more careful photography—where images are all cropped and squared at the time of data collection—the workflow can be further automated to remove all user input. This approach demands much less data entry effort at the computer, but demands greater attention to detail in the field in order to collect perfect game-board photographs. Third party Android or iOS apps, like Tiny Scanner, however, provide functionality that makes such data collection possible.

As before, the data must be read by the pre_process function, but now with the argument pre_processed=TRUE, to indicate that the photographs have already been prepared for the classifier: 
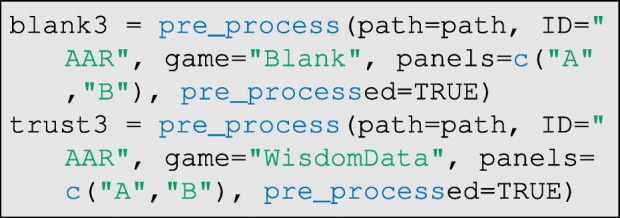


The data are again wrangled to prepare them for the classifier: 
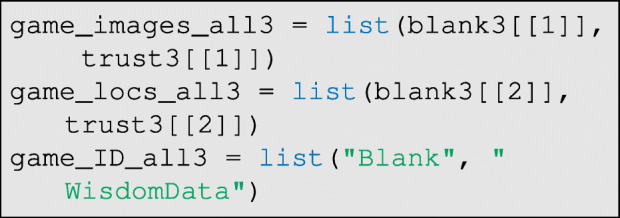
 and then the classifier is run. In this case, however, the argument pre_processed=TRUE must be set as below: 
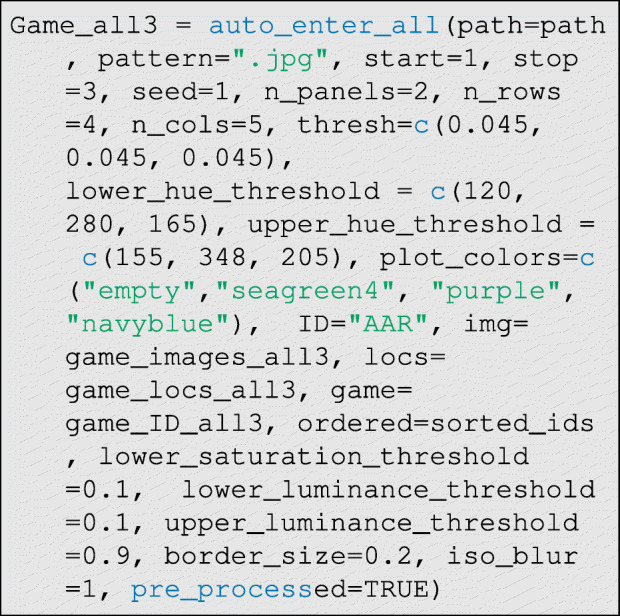


As before, the inferred classifications should be visually checked for accuracy using the check_classification function. If the classification is good, header data are then appended as before using the annotate_data function. Once all data are entered, they can be compiled into a single edge-list using the compile_data function.

## Discussion

There has been a recent drive to make tools available for researchers interested in collection of social network and relational data—e.g., Breadboard (Human Nature Lab, 2020a), Trellis (Human Nature Lab, 2020b), and Open Data Kit (ODK Team, 2020). These tools, however, are generally catered towards researchers working in areas where research participants have access to and familiarity with computer interfaces. DieTryin offers a low-cost alternative for socio-relational data collection, and caters to a different sub-field of researchers—especially cross-cultural psychologists and anthropologists—working in areas where data quality and validity may be improved by employing technologically simpler research tools—i.e., using physical photograph rosters, rather than tablet computers—to collect data. Nevertheless, DieTryin streamlines the data collection, entry, and cleaning process, through the use of high-tech functionality on the ‘back end,’ where machine learning algorithms are used to automatically code and validate edge-list data from photographs of token allocations. It is our hope that DieTryin will contribute to the compilation of a rich corpus of cross-cultural socio-relational data, and will help to alleviate some of the validity concerns associated with the use of name-generator-based methodologies.

### Building richer cross-cultural databases

Even comparatively simple cross-cultural studies of behavioral variation in classical economic games, like the Dictator and Ultimatum Games (e.g., Henrich et al.,, 2001; Henrich et al.,, 2005), have had a profound impact on our understanding of human behavior, cognitive processes, and cultural variation. New methodologies for conducting networked-structured economic games with roster-based methods, e.g., as introduced by Rucas et al., (2010) and Gervais (2017), have the potential to further extend cross-cultural studies of human behavioral variation and unpack the effects of focal, alter, and dyadic characteristics. The data generated under these methods are thus richly informative, reflecting both positive and negative ties between all community members at a dyadic level, and are broadly applicable to tests of theory in fields as diverse as behavioral economics, cross-cultural psychology, and evolutionary anthropology (Pisor et al., 2020). Although network-structured economic games generally, and RICH games specifically, have been used primarily by anthropologists studying rural social networks (Rucas et al., 2010; Gervais, 2017; Pisor et al., 2020), they can just as easily be deployed to study social relations in other bounded communities, like classrooms, sport teams, or urban neighborhoods.

These games, however, have not, as of yet, been applied across anthropological and psychological study sites as broadly as classical economic games (e.g., Henrich et al.,, 2001, 2005; Purzycki et al.,, 2016), presumably due to the difficulty of scaling roster-based social-network methods. The time burden of *in situ* data collection with paper and pencil—and on site data entry with standard, spreadsheet-based methods—scales with the square of sample size and rapidly becomes prohibitive. In this paper, we have introduced an R package that dramatically reduces the time burden of data collection and entry—hopefully stimulating wider use of RICH games and associated dyadic data collection methods across study sites. Moreover, by reducing the amount of time needed to collect, enter, and process data, DieTryin allows for more questions to be asked to respondents during a given interview—permitting collection of important control variables. Reducing the time burden of data collection also facilitates longitudinal study designs—permitting study of how dyadic game behavior changes with time and with time-varying covariates.

### Validity

A growing literature has called into question the reliability and validity of some traditional survey methods for measuring social networks—especially the name-generator method (Bernard et al. 1982; Bernard et al. 1984; Brewer, 2000; Krackhardt and Kilduff, 1999). That is, doubt has arisen about whether these tools: (i) indeed capture what the researcher believes that they do, (ii) yield consistent results if and when repeated, and (iii) lead to well-founded inferences (O’Reilly, 1988). Although self-reports of social relationships are commonly used (Kashy & Kenny, 1990; Romney & Weller, 1984; Moody & et al. 2007), an individual’s perception of, or statements about, their social ties are not necessarily impartial or accurate accounts of their social world (Krackhardt, 1987; Freeman, 1992).

Several important biases occur when using such measurements. For example, individuals may preferentially remember and nominate those with desirable qualities or high positions within their social group (Marin, 2004). Likewise, individuals are at times unable to accurately remember their interactions (Bernard et al., 1984; Brewer, 2000; Bell et al., 2007), and question/roster order effects can impact responses, especially if the data collection burden leads to respondent fatigue or boredom (Pustejovsky & Spillane, 2009).

As such, self-report data collection is not a replacement for direct observational data collection. For example, spot-check (e.g., Borgerhoff et al.,, 1985; Koster et al. 2013) time allocation data provide a more accurate representation of true time budgets than simple self-reports, which are prone to exaggeration, limited recall, and even self-deception. There is a general awareness of the benefits of collecting observed behaviors (e.g., ethnographically documented food transfers), self-reported behaviors (e.g., transfer ties as recalled by respondents), hypothetical behaviors (e.g., who respondents claim they would visit for a small loan or advice), and experimental behaviors (e.g., transfers in a network-structured economic game), as each method has its own strengths and weaknesses—but in unison, they help cross-validate one another. DieTryin is not a solution to all validity issues associated with collection of self-report data, but it functions to allow full roster data collection. This minimizes many validity concerns associated with data collected using the name-generator method. Specifically: (i) recall bias is attenuated by providing a visual prime for each community member, (ii) record linkage and de-duplication issues (Steorts et al. 2016) associated with post-processing of name-generator-based data are bypassed through the use of a full-community roster, and (iii) the speed of name-generator-based collection is maintained, reducing respondent fatigue.

### Conclusion

While it is broadly acknowledged that roster-based designs for collecting social network data provide more reliable data than free-recall name-generator-based approaches, the logistical challenges of collecting such data—such as time burden and participant fatigue—have prevented widespread use of such designs. In this paper, we introduce the DieTryin R package, which streamlines the process of collecting and managing roster-based social network data, making the approach as, if not more, feasible than free-recall name-generator designs. DieTryin expedites standardized, reproducible, and robust collection and curation of social-relational data in ecologically-valid contexts. It allows researchers across the social sciences to obtain comparable data that can be used to test broad-ranging questions across a wide array of populations.
